# Protein-peptide molecular docking with large-scale conformational changes: the p53-MDM2 interaction

**DOI:** 10.1038/srep37532

**Published:** 2016-12-01

**Authors:** Maciej Pawel Ciemny, Aleksander Debinski, Marta Paczkowska, Andrzej Kolinski, Mateusz Kurcinski, Sebastian Kmiecik

**Affiliations:** 1 University of Warsaw, Faculty of Chemistry, Warsaw 02-093, Poland; 2 University of Warsaw, Faculty of Physics, Warsaw, 02-093, Poland

## Abstract

Protein-peptide interactions are often associated with large-scale conformational changes that are difficult to study either by classical molecular modeling or by experiment. Recently, we have developed the CABS-dock method for flexible protein-peptide docking that enables large-scale rearrangements of the protein chain. In this study, we use CABS-dock to investigate the binding of the p53-MDM2 complex, an element of the cell cycle regulation system crucial for anti-cancer drug design. Experimental data suggest that p53-MDM2 binding is affected by significant rearrangements of a lid region - the N-terminal highly flexible MDM2 fragment; however, the details are not clear. The large size of the highly flexible MDM2 fragments makes p53-MDM2 intractable for exhaustive binding dynamics studies using atomistic models. We performed extensive dynamics simulations using the CABS-dock method, including large-scale structural rearrangements of MDM2 flexible regions. Without a priori knowledge of the p53 peptide structure or its binding site, we obtained near-native models of the p53-MDM2 complex. The simulation results match well the experimental data and provide new insights into the possible role of the lid fragment in p53 binding. The presented case study demonstrates that CABS-dock methodology opens up new opportunities for protein-peptide docking with large-scale changes of the protein receptor structure.

The development of peptide therapeutics is a rapidly expanding field of rational drug design strategies. New experimental as well as theoretical approaches are constantly being developed. It is mainly due to the recent successes of peptide-based therapies and the fact that peptides have a number of advantages over conventional small molecule drugs, such as high selectivity, low toxicity and smaller potential for adverse effects^1^.

The protein-peptide binding process frequently involves significant conformational rearrangements of protein receptor and peptide chains. Efficient treatment of these large-scale changes remains one of the major challenges for molecular docking^2^. The flexibility of protein targets is usually neglected or very limited during docking. The state-of-the-art tools for protein-peptide docking are dedicated to exploration of peptide flexibility rather than flexibility of the receptor^3^,^4^,^5^,^6^,^7^. Incorporation of large structural changes of protein targets in the explicit docking approach remains too computationally demanding for classical modeling tools^2^. This problem can be overcome by reducing the level of protein representation from all-atom to coarse-grained^8^. Rosetta^9^ and CABS-dock^10^ coarse-grained-based methods now appear to be the most effective tools that allow for large-scale protein motions during explicit peptide docking^11^,^12^,^13^, as outlined in the recent review on protein flexibility in drug design^2^.

In this work, we use the CABS-dock method for the molecular docking of the complex that plays an important role in cancer biology: the p53-MDM2 system^14^. The p53 protein is a transcription factor involved in the regulation of cellular processes and widely known due to its tumor suppressing action. MDM2–a natural negative regulator of p53–has recently been gaining increasing attention because of its role in the MDM2-p53 feedback loop whose distortion may be the cause of tumor progression^15^. The MDM2-p53 complex is currently intensely investigated as a potential drug target for cancer therapy^16^,^17^. A number of inhibitors of the MDM2-p53 interaction have recently been tested both *in vitro* and clinically as potential cancer therapeutics^18^,^19^,^20^,^21^,^22^,^23^,^24^,^25^.

It should be noted that the details of molecular MDM2-p53 interactions are not fully understood, mainly because of the significant flexibility of certain parts of the MDM2 receptor structure^14^. Recent reports suggest an important role of disordered regions of the MDM2 protein in complex formation^18^,^19^,^20^,^21^,^22^,^23^,^24^,^25^,^26^. Experimental data suggest that the interaction starts with binding and folding of the p53 terminal part together with MDM2 conformation change from “closed” to “open”^27^,^28^,^29^,^30^. The N-terminal flexible fragment of the MDM2 protein that covers the hydrophobic binding cleft in the “closed” state is referred to as a “lid”^31^. Therefore, MDM2-p53 docking appears to be a multilevel, dynamic process that involves a number of transient intermediate states^15^,^31^,^32^.

Here, we present and discuss the results of our prediction of the binding mechanisms and the resulting structures of the MDM2-p53 complex. To our best knowledge, the previous simulations of this complex were limited to too short simulation timescales (see Discussion) and/or shortened variants of MDM2 that excluded entire or significant portions of the highly flexible regions^25^,^26^,^31^,^32^,^33^,^34^. In the modeling procedures, we have not used any information on either the docking site or the peptide structure in the complex. Moreover, during docking simulation the disordered regions of the receptor and the p53 peptide were treated as fully flexible. Again, to our knowledge, this had not been pursued before, most likely due to the extremely large computational cost required by the conventional all-atom modeling approaches. As we present here, our approach enables exhaustive simulations of the entire system in an explicit docking procedure. The efficient treatment of p53 and the MDM2 lid region as fully flexible during docking has led to qualitatively new and interesting results on the lid role in p53 binding and complex stabilization.

## Methods

### CABS-dock method

The modeling was performed using the CABS-dock web server for flexible protein-peptide docking (freely available at http://biocomp.chem.uw.edu.pl/CABSdock/) without a priori knowledge about the binding site. CABS-dock enables full flexibility of the peptide structure and large-scale flexibility of protein fragments during the blind search for a binding site. Detailed description of the CABS-dock server and its benchmark tests have been recently described^10^. Several examples of CABS-dock applications and extensions have been also described in a recent publication^11^.

The CABS-dock modeling method is based on the efficient simulation scheme of coupled binding and folding of a peptide using the CABS coarse-grained model (described in detail some time ago^35^ and recently discussed in the review^8^). CABS is a well-established modeling tool extensively tested in many applications, including the folding and binding mechanism of an intrinsically disordered peptide^36^, folding mechanisms of globular proteins from the denatured to the folded state^37^,^38^,^39^, simulation of protein dynamics, near-native structure fluctuations^40^,^41^ and protein structure prediction^42^,^43^. In the CABS-dock automated protocol^10^,^11^, CABS coarse-grained simulation is merged with the all-atom local optimization of selected reconstructed models.

### Input data

The MDM2-p53 interaction is stabilized mainly by the N-terminal MDM2 segment (residues 25–109) and a short, helical p53 region (residues 19–26, often isolated as a peptide in experimental studies of the MDM2-p53 interaction). The MDM2-p53 X-ray complex structures are available in PDB under the codes 1T4F and 1YCR. The NMR structure of the unbound (peptide-free) MDM2 is available under the code 1Z1M.

As the input receptor structure, we used the first model from the NMR ensemble of structures of unbound MDM2 (default setting of the CABS-dock server for NMR ensembles).

**Figure 1:**
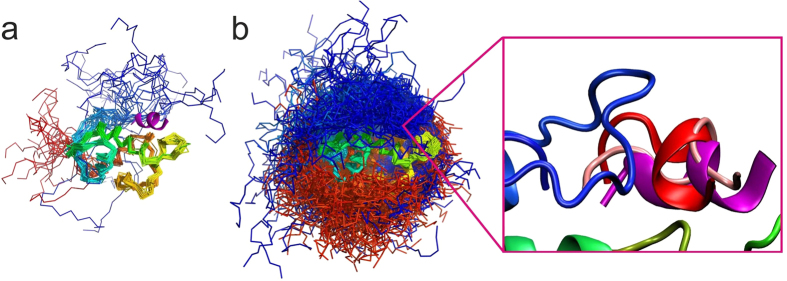
Comparison of MDM2-p53 experimental structures (**a**) and CABS-dock simulation models (**b**). Panel (a) shows NMR structures of the MDM2 receptor in the unbound form (colored from blue to red, PDB ID: 1Z1M) together with the experimental X-ray structure of the p53 peptide (colored in magenta, PDB ID: 1T4F; note that the X-ray structure of the MDM2 is highly similar to the ordered portion of its NMR ensemble presented in the figure). Panel (b) shows an ensemble of 10,000 CABS-dock simulation models of the MDM2 receptor (left), and predictions of the peptide structure (right), together with the experimental p53 peptide structure (colored in magenta). Two peptide predictions are shown: the top scored (pink color, peptide-RMSD: 3.74 Å) and the closest to the experimental structure (red color, peptide-RMSD: 2.67 Å).

The only input data about the p53 peptide was its amino acid sequence of 9 residues: RFMDYWEGL (no information about the p53 secondary structure was used). In the CABS-dock simulations with default settings, the peptide structure is fully flexible and the receptor structure is kept near the input conformation using soft distance restraints. The restrains allow small fluctuations of the receptor backbone in the range of 1 Ångstrom and, consequently, large fluctuations of the side chains. On top of the default settings, additional flexibility may be assigned to selected fragments by ignoring the distance restraints for these fragments^10^,^11^. We used this option to assign full flexibility to two MDM2 regions. Based on the analysis of the variability of the structures resulting from the NMR experiments (see Fig. 1a), these two motile regions are: the N-terminal region (residues 1–27) and the C-terminal region (residues 106–119). Thus, the unrestrained MDM2 fragments (of significant length: 27 and 14 residues) were allowed to undergo large-scale movements. What is important to note, a starting conformation of protein receptor fragments with assigned “full flexibility” has no impact on modeling results because of the extremely efficient sampling of the conformational space of such receptor fragments (given the default simulation length). An exception may be starting structures with the disordered fragments entangled with the rest of the receptor structure, which is not the case here.

### Analysis of results

To evaluate the modeling results, we use “peptide-RMSD”, which is defined as the root mean square deviation of the C-alpha atoms in the peptide model from their experimental positions after superimposition of the receptor structures of the compared complexes. For selected models, we also calculate percentage of the native contacts. The percentages are derived using contact maps from the CocoMaps server^44^ with a cut-off distance value of 8 Å. For peptide-RMSD and contact map calculations, we used the crystallographic structure of the MDM2-p53 complex (PDB: 1T4F) as the reference “native” structure.

## Results

### General overview

The standard CABS-dock web server procedure generates 10,000 model structures of the protein-peptide complex. In this study, we used clustering based on the RMSD of the entire protein-peptide complex. The resulting structures are grouped in clusters of similar complexes and ranked according to cluster size from the largest to the 10th largest. Ten top ranked CABS-dock models (representatives of the 10 most numerous clusters) are discussed below.

The NMR ensemble of unbound MDM2 structures and 10,000 CABS-dock generated structures, respectively, are presented in Fig. 1a and b. The significant flexibility of the disordered regions of the MDM2 protein is represented in the NMR ensemble (Fig. 1a). In comparison to the NMR ensemble (consisting of 24 models), the set of 10,000 CABS-dock models shows a significantly more abundant set of different arrangements of disordered MDM2 ends (Fig. 1b). During the docking simulation, the flexible N- and C-termini remained disordered as suggested by the experimental results for MDM2 dynamics^27^,^28^,^29^,^30^. As presented in the close-up frame (Fig. 1b), the N-terminal region of the protein may interact with the peptide in the bound form.

**Figure 2:**
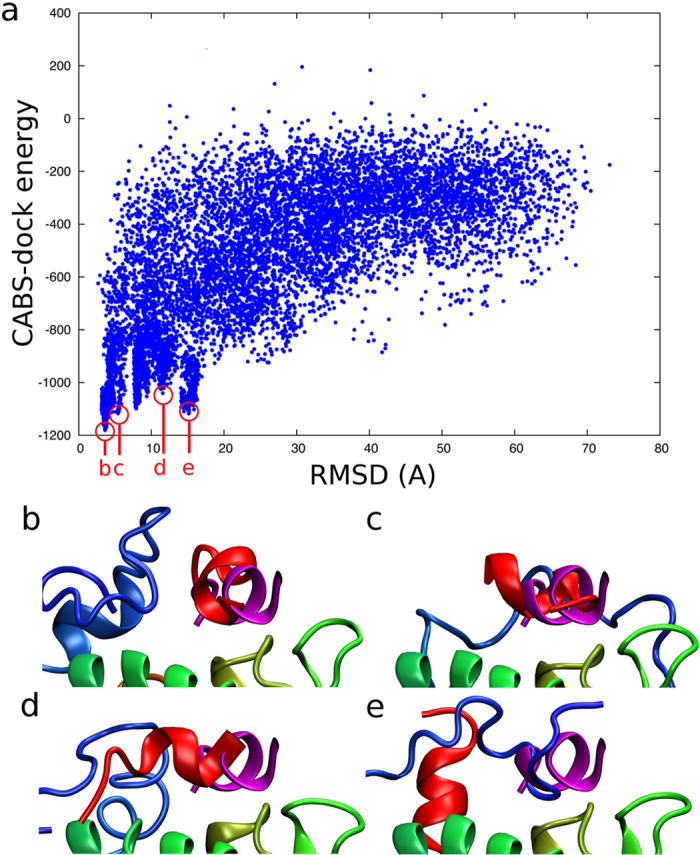
Peptide-RMSD versus CABS-dock energy and example top ranked models obtained in CABS-dock docking. Panel (a) shows the peptide-RMSD vs. CABS-dock energy graph for 10,000 CABS-dock models. The markers indicate the best models produced in the docking. The structures we obtained represent the p53 peptide bound close to the binding site and the receptor in the “open” conformation (**b**–**d**) and also models with the N-terminal lid docked in the binding site - the receptor in “closed” conformation (**e**). The receptor protein is colored from blue to red (partially visible, the lid is colored in blue), the peptide model is colored in red and the X-ray structure of the peptide is shown in magenta. The peptide-RMSDs of the models were (**b**) RMSD = 3.74 Å (1st ranked model), (**c**) RMSD = 4.36 Å (2nd ranked model), (**d**) RMSD = 11.26 Å (3th ranked model), and (**e**) RMSD = 15.79 Å (8rd ranked model).

First seven top-ranked structures present the peptide bound in the proximity of the binding site, whereas the remaining three show the receptor in a “closed” state with the lid bound to the binding site. The top ranked p53 peptide models are presented and compared with the X-ray structure in Fig. 1b (1st ranked model) and Fig. 2b–e (1st, 2nd, 3rd and 8th ranked models). The analysis of these structures shows that the procedure not only managed to predict the binding site of MDM2 but also partially reconstructed the alpha-helical structure of the bound peptide. The 1st ranked model is characterized by the peptide-RMSD value of 3.74 Å, and it reproduced 60% of the native contacts. The analysis of the full set of 10,000 models showed the model with the lower peptide-RMSD value of 2.67 Å (see Figs 1b and 2d); however, with the interaction pattern slightly less accurate – 54% of the native contacts were present in this structure.

### Contact map analysis

As demonstrated in our recent study^36^, the CABS-dock docking procedure can be effectively used in the characterization of transient protein-peptide encounter complexes and investigation of binding mechanisms for disordered proteins. The interactions of highly dynamic complexes can be conveniently analyzed using contact maps derived from the CABS-dock simulations^45^ (the contact maps are automatically calculated using centers of the mass of the side-chains and stored in zip files available for download for each job on the CABS-dock server).

**Figure 3:**
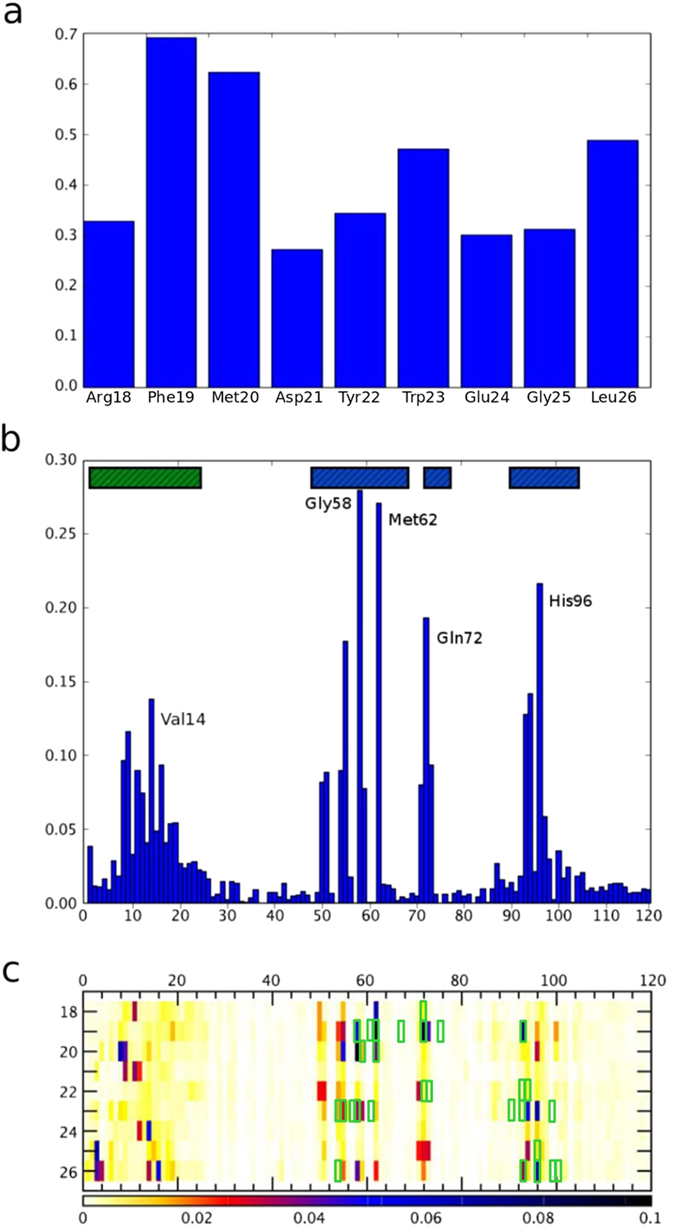
Protein-peptide contact analysis. All panels (a–c) show averaged results from CABS-dock docking (over 10,000 models). Panel (a) presents frequencies of contact formation by p53 peptide residues, histogram (**b**) presents frequencies of contact formation by MDM2 residues (the green and blue bars mark the lid and binding site regions of the receptor, respectively). The binding site and the lid residues that contact the peptide most frequently are marked. Panel (c) shows a contact frequency map between residues from MDM2 and the p53 peptide together with native contacts marked in green for reference.

We analyzed intermolecular (protein-peptide) and intramolecular (within the protein receptor) contacts focusing on the dynamics of the N-terminal lid fragment. Analysis for the observed intermolecular contact frequency together with the contact map is shown in Fig. 3. The detailed information on inter- and intramolecular contact frequencies is shown in Supplementary Materials (Supplementary Tables S1 and S2).

First of all, we examined the contact-forming residues of the peptide to find out which parts of the p53 modeling peptide are most important for its interaction with MDM2. According to the experimental results, the most important complex-stabilizing contacts are formed by three p53 hydrophobic residues: Phe19, Trp23 and Leu26. The same pattern is found in our results as increased contact frequencies visible in Fig. 3a. Additionally, we noted an increased contact frequency for p53 residue Met20 in our simulations, which is probably the effect of neighboring with Phe19.

The fourteen MDM2 residues reported to form the binding site of MDM2 are: Met50, Leu54, Leu57, Gly58, Ile61, Met62, Tyr67, His73, Val75, His96, Ile99 and highly conserved Tyr100. Out of those, Leu54, Gly58, Ile61, Met62, Tyr67, His96, Ile99, Tyr100 have been identified as the most important according to experimental studies^15^,^32^,^46^,^47^,^48^,^49^,^50^. In our simulations, we observed the highest contact frequencies for the residues: Gly58, Met62, Gln72, His96 and increased frequencies for their neighbors (as indicated in the histogram in Fig. 3b and shown in Supplementary Table S1). Moreover, the experimental data show that MDM2 residue Gly58 is crucial for complex binding as its mutation results in a loss of bonding with p53. The significance of this residue is also strongly highlighted in our simulation results: Gly58 has the highest contact frequency found in the simulations (see Fig. 3b and Supplementary Table S1). The MD results also suggest the significance of Gln72^32^: in our study we recorded frequent contacts between the peptide and this residue.

Finally, we analyzed the protein-peptide contact map in search for the most frequently formed contacts during the docking simulation. The map (see Fig. 3c) shows the frequencies of MDM2-p53 contacts together with the crystallographic contacts indicated for comparison. The most frequent contacts detected in our models are either in close neighborhood or are the same as the native. The highest peaks were observed for two protein-peptide contacts: Gly58-Phe19 and Gln72-Phe19. p53 residue Phe19 is often reported as crucial for the p53-MDM2 interaction in experimental studies^49^,^50^,^51^,^52^. Its importance is also visible in our simulation results as a distinct contact frequency peak in Fig. 3a and c. The contacts between MDM2 Ile61, Val 93 and p53 Phe19, Trp23 which are responsible for hydrophobic interactions with the binding site show the frequency level of 0.06–0.1 in the simulation (Supplementary Table S2). In addition, we noticed contacts on a high frequency level for Leu54 and Trp23 which are reported as important for the stabilization of the p53-MDM2 complex in other simulation studies^32^.

### Lid dynamics

**Figure 4:**
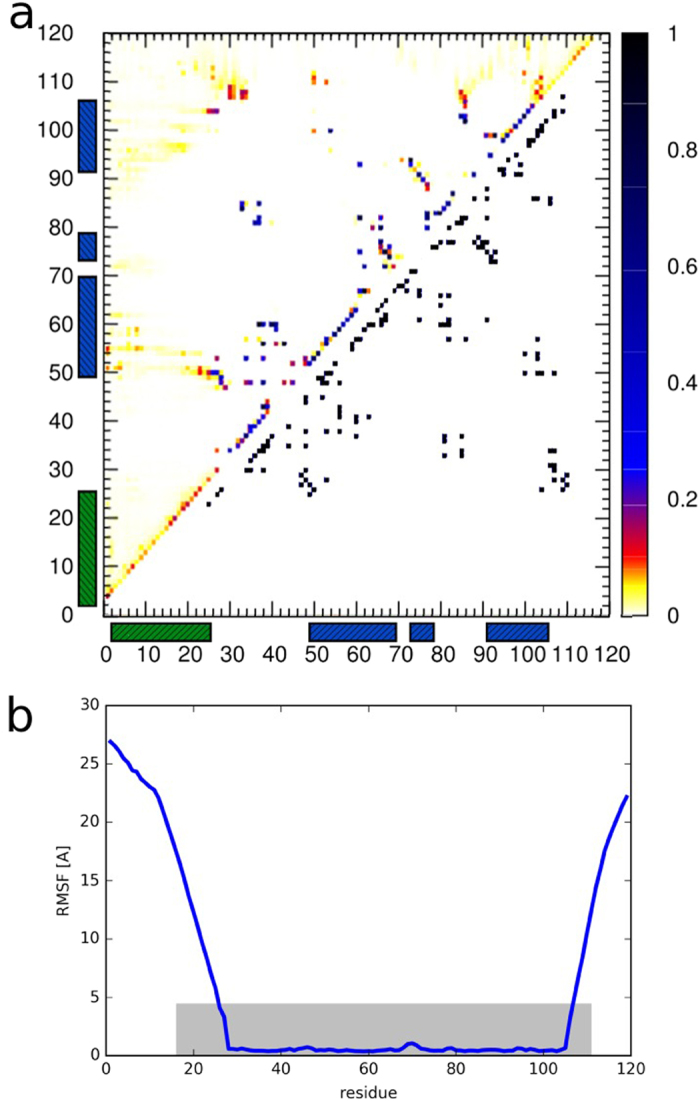
Analysis of lid dynamics. (**a**) Above diagonal - intramolecular contact map for the MDM2 protein in CABS-dock docking representing averaged frequencies of intra-protein contacts of the MDM2 protein in the docking simulation (comprising 10,000 models); below diagonal - native intramolecular contact map for the MDM2 protein based on the crystallographic structure (PDB ID: 1T4F). The green and blue bars along the axes mark the lid and binding site regions of the receptor, respectively. (**b**) RMSF (root-mean square fluctuation) averaged over the trajectory from the CABS-dock simulation (blue line). The grey rectangle shows the area investigated so far with all-atom MD (reported in refs 25,31, 32, 33, 34). The rectangle borders show the maximal RMSF value obtained and the largest MDM2 fragment included in these simulations.

We used intra-protein contact maps and RMSFs (Root Mean Squared Fluctuations) averaged over simulation trajectories to qualitatively analyze the dynamics of the flexible MDM2 segment. The resulting graphs are presented in Fig. 4 and the contact frequency values are shown in Supplementary Table S2. The intra-protein contact map (Fig. 4a) shows that the contacts between the lid and the binding site of the receptor are indeed observed in our simulations as reported in experimental studies^27^,^28^,^29^,^30^. The RMSF graph (Fig. 4b) shows clearly that the terminal regions of MDM2 undergo significant conformational changes of much higher fluctuation levels than those obtained in all-atom MD simulations^25^,^31^,^32^,^33^,^34^.

The simulation results show that the lid-peptide interaction results in a number of different structures. In Fig. 2, we show top ranked models of the p53 peptide together with different conformations adopted by the flexible lid. It either forms different arrangements in the proximity of the bound peptide (see Fig. 2a–c) or even takes its place in the binding site of the MDM2 protein (Fig. 2d).

The maps (Fig. 4a) show that during the docking the lid forms contacts with the binding site (preferably with its part formed by receptor residues 50–60). Lid residues Leu27 and Ile19 most frequently form contacts with the well-structured protein core during our simulations, which is consistent with the NMR studies^29^,^28^. Additionally, according to the these experiments^29^,^28^, the lid residues 21–24 may adopt a marginally stable p53-like helix conformation in the closed state of the receptor. A similar conclusion may be drawn from the contact map (Fig. 4a) obtained from our simulations. A region of increased frequency of near-neighbor contacts that are characteristic for a helical structure could be observed on the map for residues 21–24 of the MDM2 receptor.

The protein-peptide frequency map (Fig. 3c) has been discussed so far in the context of the receptor binding site. However, noticeable contacts are also visible in the area representing contacts between the lid and the peptide. Experimental results show significant perturbation of the MDM2 residues 16–24 that accompanies binding of the p53 peptide and may be caused by its interaction with the lid during competition for the binding site^27^,^28^,^29^,^30^. A similar competition picture can be drawn from our simulation analysis. The peaks of averaged frequencies of contact between the lid and the binding site have heights of 0.2–0.1 (Supplementary Table S2), which is comparable with the frequencies of contacts between the p53 peptide and the binding site (Supplementary Table S1).

## Discussion

In this work, we used the CABS-dock method for modeling large-scale conformational changes during p53 peptide binding to the MDM2 protein receptor. We obtained a large ensemble of near-native models with different arrangements of flexible MDM2 fragments and the p53 peptide, without using any a priori information about the binding site or the peptide structure. The accuracy (peptide-RMSD) of the best obtained model is 2.76 Å and 3.74 Å for the top ranked model. What is important to note, this accuracy level is sufficient to improve the model’s quality to below 2 Å (in terms of interface RMSD) using all-atom refinement, as presented in Raveh *et al*.^53^. Moreover, the simulation results confirmed the important role of particular residues identified in the experiment as crucial for the binding process and provided insight into the highly dynamic interactions of the MDM2 lid fragment.

To our best knowledge, investigations of MDM2 flexibility during p53 binding have been limited so far either by ignoring the entire lid fragment in the simulation system and/or too short simulation timescales: of a nanosecond^25^,^31^,^32^,^33^,^34^ or a microsecond^26^ scale. A very recent computational study, constructing Markov State Models from many independent trajectories of apo-MDM2^54^, suggests that even microsecond MD simulations of apo-MDM2 are not sufficient to adequately sample the conformational space of the flexible lid in the unbound receptor. As shown in experimental and computational studies of the MDM2 system, capturing the correct dynamics of disordered regions may be crucial for peptide binding^14^,^54^. In comparison to most of the simulation studies mentioned above, our method enabled a quantitative leap from the dynamics of side-chain fluctuations to large-scale motions of flexible MDM2 segments. As compared to more extensive simulation approaches, like presented in ref. 54, CABS-dock provides an effective yet inexpensive alternative. Thanks to an efficient multiscale docking approach, the presented CABS-dock results were produced in a matter of hours using a single CPU. This makes our method uniquely fast and, despite the applied coarse-graining, surprisingly accurate in its blind predictions.

## Additional Information

**How to cite this article**: Ciemny, M. P. *et al*. Protein-peptide molecular docking with large-scale conformational changes: the p53-MDM2 interaction. *Sci. Rep.* **6**, 37532; doi: 10.1038/srep37532 (2016).

**Publisher's note:** Springer Nature remains neutral with regard to jurisdictional claims in published maps and institutional affiliations.

## Supplementary Material

Supplementary Information
