# Supplementary material for: Protein-peptide molecular docking with large-scale conformational changes: the p53-MDM2 interaction
Source: Sci Rep. 2016 Dec 1;6:37532. doi: 10.1038/srep37532 (PMC5131342; doi:10.1038/srep37532)

Manuscript title: Protein-peptide molecular docking with large-scale conformational changes: the p53-MDM2 interaction

Authors: Maciej Pawel Ciemny[1,2,+], Aleksander Debinski[1,+], Marta Paczkowska[1], Andrzej Kolinski[1], Mateusz Kurcinski[1,*], Sebastian Kmiecik[1,*]
[1] University of Warsaw, Faculty of Chemistry, Warsaw 02-093, Poland
[2] University of Warsaw, Faculty of Physics, Warsaw, 02-093, Poland
[*] mkurc@chem.uw.edu.pl, sekmi@chem.uw.edu.pl
[+] these authors contributed equally to this work

Supplementary information

Supplementary Table S1. Protein-peptide contacts. The table presents 30 most frequent protein-peptide contacts observed in the 10,000 frames of the CABS-dock simulation.

p53 residue	MDM2 residue	Contact frequency
Ser20	Gly58	0.1378
Phe19	Met62	0.1072
Phe19	Gln72	0.0928
Phe19	Val93	0.0709
Thr18	Met62	0.0657
Trp23	His96	0.065
Ser20	Val8	0.0637
Leu26	His96	0.0628
Lys24	Val14	0.0532
Trp23	Lys94	0.0511
Leu26	Thr16	0.049
Ser20	Phe55	0.048
Phe19	Gly58	0.048
Phe19	His73	0.0433
Ser20	Pro9	0.0416
Leu26	Gly58	0.0414
Ser20	Met62	0.0374
Asp21	Asp11	0.0371
Asp21	Pro9	0.0342
Leu25	Lys94	0.0339
Thr18	Asp11	0.0337
Leu26	Val14	0.0331
Trp23	Gln59	0.0331
Leu26	Val93	0.0331
Trp23	Gly58	0.0328
Phe19	Phe55	0.0324
Leu22	Gln71	0.0309
Ser20	His96	0.0306
Leu25	His73	0.0293
Lys24	Gly12	0.0285

Supplementary Table S2. Intramolecular lid-receptor contacts. The table presents 30 most frequent lid-protein contacts observed in the 10,000 frames of the CABS-dock simulation.

MDM2 residues		Contact frequency
Leu27	Thr49	0.2189
Leu27	Met50	0.2122
Met1	Phe55	0.0825
Leu27	Pro30	0.0754
Thr26	Thr49	0.0506
Thr26	Met50	0.0436
Ile19	Arg97	0.0421
Met1	Lys51	0.039
Glu25	Met50	0.038
Met6	Lys94	0.0317
Glu25	Thr49	0.0305
Ile19	Gln112	0.0277
Thr26	Arg29	0.0222
Leu27	Tyr28	0.0179
Thr4	Lys94	0.0173
Gln24	Met50	0.0165
Val8	Phe55	0.0163
Ile19	Thr101	0.0155
Glu23	Lys51	0.0149
Thr26	Pro30	0.0146
Glu23	Gln112	0.014
Ser7	Lys94	0.0125
Thr15	Glu95	0.0123
Ser22	Lys51	0.012
Thr4	His73	0.0119
Val8	Glu95	0.0119
Thr4	Gln72	0.0118
Thr16	Glu95	0.0116
Asn5	Lys94	0.0114
Ser17	Arg97	0.0111

Supplementary Figure S1. CABS-dock energy versus the fraction of native receptor-peptide contacts (N_C) observed in the 10,000 frames of the CABS-dock simulation. The contacts are derived using cut-off distance value of 8Å (based on center of the mass positions of the side chains). The graph shows that the structures with higher fraction of native contacts tend to lower CABS energy values.

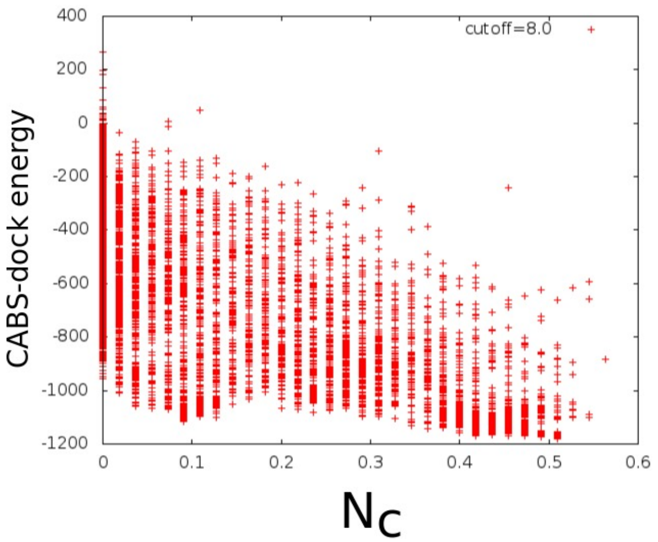

Supplement: Supplementary Information [file srep37532-s1.pdf]
